# Evaluation of protective immune responses induced by DNA vaccines encoding *Echinococcus granulosus* EgM123 protein in Beagle dogs

**DOI:** 10.3389/fvets.2024.1444741

**Published:** 2024-09-25

**Authors:** Ning Wang, Jinwen Xian, Pengpeng Zhao, Wenqing Zhao, Na Pu, Xinyue Jia, Yanyan Zhang, Xinwen Bo, Zhengrong Wang

**Affiliations:** ^1^State Key Laboratory of Sheep Genetic Improvement and Healthy Production, Shihezi, China; ^2^Institute of Animal Husbandry and Veterinary Medicine, Xinjiang Academy of Agricultural and Reclamation Science, Shihezi, China; ^3^College of Animal Science and Technology, Shihezi University, Shihezi, China; ^4^College of Animal Science and Technology, Tarim University, Xinjiang, China

**Keywords:** *Echinococcus granulosus*, EgM123, DNA vaccine, definitive-host, worm burden

## Abstract

**Introduction:**

Echinococcus granulosus, known as cystic echinococcosis, is a prominent zoonotic parasitic disease of significant global concern. The definitive hosts serves as the primary reservoir for the transmission of echinococcosis, as well as a main factor in the prevention and control of the disease. Unfortunately, there is currently no commercially available vaccine for these hosts. Nevertheless, DNA vaccines show potential as a feasible strategy for the control and management of parasitic diseases.

**Methods:**

In this study, the EgM123 antigen was selected for its well-documented immunogenic properties to develop a DNA vaccine aimed at combating *E. granulosus* infection in canines.

**Results:**

The results showed a marked increase in IgG levels in the group vaccinated with pVAX1-EgM123 DNA compared to the PBS group. Additionally, the cytokines IL-1, IFN-*γ*, IL-4, and IL-6 were significantly upregulated in the pVAX1-EgM123 DNA vaccine group. Furthermore, in comparison to the PBS control group, the EgM123 DNA vaccine group exhibited a notable 87.85% reduction in worm burden and a 65.00% inhibition in segment development.

**Discussion:**

These findings indicate that the pVAX1-EgM123 DNA vaccine shows promising immunogenicity, successfully eliciting a targeted immune response in canines. Moreover, it significantly diminishes the worm burden and hinders the progression of tapeworms in the pVAX1-EgM123 DNA vaccine group. These findings suggest that the pVAX1-EgM123 DNA vaccine holds promise as a potential candidate vaccine for combating *E. granulosus* infection in dogs.

## 1 Introduction

Cystic echinococcosis (CE) is a zoonotic parasitic disease caused by the larvae of *E. granulosus* in various animal species, including cattle, sheep, pigs, and humans. Currently, the global prevalence of this disease affects approximately 1 million individuals (1). In certain geographical areas of South America, Africa, and Asia, the disease exhibits a prevalence rate that can reach up to 10% of the population. Statistical data reveals that the occurrence of sheep cystic echinococcosis, as detected in slaughterhouses situated in regions of South America that are considered high-risk, ranges from 20 to 95%. China is one of the countries with high incidence (2–4). Echinococcosis exhibits a high prevalence across 20 provinces in China, with seven provinces in Northwest China being recognized as endemic areas. These regions pose a significant threat to the well-being of approximately 70 million individuals and 60 million livestock. Furthermore, the annual economic repercussions of this disease can amount to a staggering 3 billion yuan (5, 6). CE has been included among the five parasitic diseases outlined in the “Healthy China 2030” initiative. Furthermore, it is among the six primary infectious diseases for which treatment is provided free of charge in China (2). *E. granulosus* can be transmitted across different hosts, and the target host should be designated as the main factor in the design of vaccines (7). Given that *E. granulosus* exhibits a multi-host lifecycle, the primary objective of vaccine development for this parasite is to disrupt its distinctive circulation chain, thereby effectively mitigating the dissemination of *E. granulosus* (8).

The EgM family comprises a group of soluble proteins abundant in cysteine, which are associated with the growth and development of *E. granulosus*. Zhang and colleagues effectively identified the EgM family from protoscolece (PSC) through the utilization of RT-PCR, with members including EgM4, EgM9, and EgM123. Subsequent analyses involved subcloning and prokaryotic expression of EgM4, EgM9, and EgM123, followed by immunization of canines with the purified proteins (9, 10). The findings demonstrated that, in comparison to the control group, the three recombinant proteins exhibited the ability to impede worm growth, particularly in terms of inhibiting ovulation. Moreover, the impact of rEgM123 was notably more pronounced (10). In 2018, Zhang and his team conducted a study in which dogs were immunized with recombinant EgM4, EgM9, and EgM123 proteins. The study findings demonstrated that rEgM9 had a more significant inhibitory effect on the development and ovulation of worms, while rEgM123 greatly reduced the worm burden in the dogs (11). These results provide further support for the potential suitability of EgM family antigens as DNA vaccine antigens in the future for combating *E. granulosus* infection.

The DNA vaccine involves the introduction of exogenous genes (DNA) that encode specific antigen proteins into regulatory elements responsible for gene expression. Animals are then immunized through various immune pathways to facilitate the expression of these genes. Consequently, the expressed antigen elicits targeted cellular and humoral immune responses, thereby serving as a therapeutic and preventive measure against diseases (12). The structure of DNA vaccines encompasses target genes and expression vectors (13). In contrast to protein vaccines, the efficacy of DNA vaccines relies on their ability to penetrate the cytoplasm and stimulate antigen expression *in vivo*, thereby facilitating antigen presentation and T cell recognition on major histocompatibility complex (MHC) molecules. Extensive evidence has demonstrated the potential of DNA vaccines in the prevention and treatment of infectious diseases, including cancer, parasites, autoimmune disorders, and allergic diseases, in diverse animal models (14, 15). Parasitic diseases encompass a significant category of infectious diseases that pose a substantial threat to human well-being. The advent of DNA vaccines presents a promising avenue for the exploration of parasitic vaccines. This study details the development and administration of the pVAX1-EgM123 DNA vaccine in canine definitive hosts, along with an initial assessment of its immunogenicity. These findings contribute to the theoretical framework for the progression of DNA vaccines aimed at targeting *E. granulosus* in dogs.

## 2 Materials and methods

### 2.1 Experimental dogs and protoscoleces of *Echinococcus granulosus*

The animals used in this experiment are 8-months-old Beagle dogs, purchased from Sichuan Musk Breeding Institute Beagle Breeding Center. Before purchase, dogs were vaccinated twice with Eurican DHPPI-Ll vaccine and once with Five Star live vaccine. Three times for deworming control, the deworming drug was: levamisole. The protoscoleces used in the experiment were collected from fresh livers of sheep (10) suffering from echinococcosis in Xinjiang Urumqi cattle and sheep slaughterhouse.

This study was reviewed and approved by the Care and Use of Laboratory Animals of the Xinjiang Academy of Agricultural and Reclamation Sciences (Shihezi, China) (XAARS; Approval no. 2019–012, April 9, 2019). All animals were handled in strict accordance with the animal protection laws of the People’s Republic of China (a draft animal protection law was released on September 18, 2009) and the National Standards for Laboratory Animals in China (executed on January 5, 2002).

### 2.2 Construction and preparation of pVAX1—EgM123 plasmid

The total RNA of *E. granulosus* protoscoleces was extracted using TRIzol reagent (Invitrogen) according to the manufacturer’s instructions. First-strand cDNA were synthesized using a Reverse Transcription System Kit (Invitrogen). The complete open-reading frame (ORF) of EgM123 was obtained by RT-PCR amplification from cDNA using specific primers. Forward primer: 5′ -CGG GAT CCA TGG AAC CAG TGA ATT TTG-3′ (*BamH* I), Reverse primer: 5′ -CCC TCG AGT TAT TTC GTC TTT AAG GCA C-3′ (*Xhol* I). The obtained PCR product of EgM123 gene was inserted into the pMD-19 T Vector (TaKaRa), generating pMD-19 T-EgM123. The EgM123 fragments were cleaved by *BamH* I and *Xhol* I from pMD-19 T-EgM123 and then subcloned into pVAX I (Invitrogen), which was cleaved by *BamH* I and *Xhol* I, by T4 DNA ligase to generate pVAX-EgM123 plasmid. The recombinant plasmids were identified by PCR, double restriction enzyme digestion, and sequencing. The positive plasmids were purified from transformed *Escherichia coli* DH5α cells by anion exchange chromatography (EndoFree plasmid giga kit, Qiagen Sciences, MD, United States) according to the manufacturer’s instructions. The concentrations of plasmids were determined using spectrophotometer at OD260 and OD280. Then, sterile phosphate-buffered saline (PBS) was added to the plasmid DNA solution (in elution buffer) to achieve a final concentration of 1 mg/mL, and the solution was stored at −20°C until use.

### 2.3 Expression of EgM123 in HEK-293 cells

The endotoxin-free plasmids pVAX-EgM123 and the empty vector pVAX were transfected into HEK-293 cells using lipofectamine 3000 (Invitrogen) for gene expression analysis. Western blot was performed to detect the expression of EgM123 protein. Cell lysates were prepared by collecting cells at 48 h post-transfection and lysing them with RIPA buffer (Beyotime) on ice for 30 min. The lysates were then centrifuged at 12,000 × g for 3 min, and the resulting supernatants were combined with protein loading buffer. For Western blot, cells were treated with RIPA cell lysis buffer and equal amounts of total protein were separated on 10% SDS-PAGE gels and thereafter transferred to PVDF membranes. The membranes were then blocked in 5% skim milk at room temperature for 2 h and incubated with the primary antibody (*E. granulosus* positive polyclonal antibodies were collected from dogs infected with *E. granulosus* in our lab (16, 17); 1:200 dilution) at 37°C for 1 h. After three washes with TBST buffer, the membranes were incubated with the secondary detecting antibody (HRP-labeled goat anti-dog IgG (H + L) secondary antibody; 1:800 dilution) at 37°C for 1 h. The conjugated substrate was detected using 3, 3’-Diaminobenzidine tetrahydrochloride reagent (Sigma, United States).

### 2.4 Immunization and challenge

Thirteen Beagle dogs were randomly divided into four groups, Group I was vaccinated with PBS as a control group (*n* = 3), Group II was vaccinated with the adjuvant Quil A (Sigma) (*n* = 3), and Group III was vaccinated with pVAX1 group (*n* = 3) and Group IVwas vaccinated with pVAX1-EgM123 DNA vaccine group (*n* = 4). All experiments Beagle dogs were immunized through pelvic semitendinosus or semimembranous muscle. The pVAX1-EgM123 DNA vaccine group and the pVAX1 group were immunized with 500 μg of plasmid DNA and 100 μg of QuilA dissolved in 500 μL sterile PBS, the QuilA group were immunized with 100 μg of QuilA dissolved in 500 μL sterile PBS and the PBS group were immunized with 500 μL sterile PBS. All the experiments Beagle dogs were administered three injections with the same dose at 0d (prime), 14d (1st boost) and 28d (2nd boost), with 14 days interval (Table 1).

**Table 1:** Animal experiment design.

Group	Quantity (*n*)	Immune plasmid	Immunization dose	Immunization method	Times of immunizations	Intervals(day)
PBS	3	–	–	Pelvic semitendinosus Semimembranosus	0d (prime) 14d (1st boost) 28d (2nd boost)	14
QuilA	3	–	500 ug			
pVAX1	3	pVAX1	500 ug			
pVAX1-EgM123	4	pVAX1-EgM123	500 ug			

Fourteen days after the last immunization (42d), all experiments Beagle dogs were challenged with 100,000 protoscoleces. For antibody and cytokine analysis, blood samples were collected from all experiments canines in forelimb vein at 0d, 14d, 28d and 42d after immunization, 10d, 20d and 28d after Challenge, the blood samples were centrifuged at 3000 rpm for 5 min, collected serum samples were stored at −80°C. Twenty eight days after Challenge, all experiments Beagle dogs were euthanized by intravenous injection of pentobarbital and the worms in small intestine were harvested for analysis of parasite reduction rate and the inhibitory effect on the growth and development of worms.

### 2.5 Detection of antibody by ELISA

The ELISA conditions were optimized by checkerboard titration of rEgM123 antigen and sera. The purified rEgM123 protein (5 μg/mL) was diluted in 0.1 M carbonate buffer (pH 9.6). The ELISA plates were coated with the diluted antigen solution overnight at 4°C. After washing with PBST, the plates were incubated with 5% skim milk for 2 h at 37°C. The wells were washed thoroughly and incubated with 100 μL of serum samples (1:80) in PBST at 37°C for 1.5 h. After washing, the HRP-labeled rabbit anti-dog IgG (1:3000; Solarbio, Beijing, China) were added to the plates and incubated at 37°C for 1.5 h. Then, the wells were washed again and incubated with the substrate 3,3′,5,5’-Tetramethylbenzidine (TMB) (Tiangen, Beijing, China) at 37°C for 15 min. Finally, the reaction was stopped with 100 μL of 1 M H_2_SO_4_ and the optical density at 450 nm (OD450) was determined.

### 2.6 Detection of cytokines in dogs using ELISA

The immune stimulation effect of the pVAX1-EgM123 DNA vaccine on dogs was evaluated by quantitative ELISA at day 28 post challenge. Dog cytokine ELISA Quantitation Kits (Jianglaibio, Shanghai) were used to quantify IL-1, IL-4, IL-5, IL-6 and IFN-*γ*, respectively. An ELISA strip in its aluminum foil bag was left at to equilibrate at room temperature for 60 min. The strip was removed from the bag and the standard sample well (dog sera). Each well received 50 μL of dog sera at different concentrations, respectively. Then, 100 μL of HRP labeled antibody was added to each well. The reaction wells were sealed with a sealing film and incubated at 37°C for 60 min. The liquid was then discarded and the plate was patted dry using absorbent paper. Each well was filled with 350 μL of PBST and left to strand for 1 min. The detergent was removed, the plate was patted dry using absorbent paper, and the detergent washing step was repeated five times. Each well was then incubated with 50 μL of substrate A and 50 μL of substrate B at 37°C for 15 min. Then, 50 μL of termination solution was added into each well, and the OD value of each well was measured at 450 nm within 15 min.

### 2.7 The worms burden and the segments development

In all experiments, Beagle dogs were euthanized and necropsied to collect and count worms as previously described (18, 19). Thirty worms were chosen randomly from each experimental group and the sizes of developed (≥4 segments) vs. underdeveloped (≤3 segments) worms were determined (10).

### 2.8 Data analysis

All data were analyzed using SPSS 22.0 software (version 22.0; IBM Corp, Armonk, NY, United States) and one-way analysis of variance (ANOVA). Differences between tested groups were considered significant if the *p* value was ≤0.05, and this is indicated in the figures by asterisks (*p < 0.05; **p < 0.01; ***p < 0.001). All experiments were repeated a minimum of three separate times. Graphs were generated using GraphPad Premier 6.0 software package (GraphPad Prism, San Diego, California, United States).

## 3 Results

### 3.1 Identification of eukaryotic vector construction

**Figure 1:**
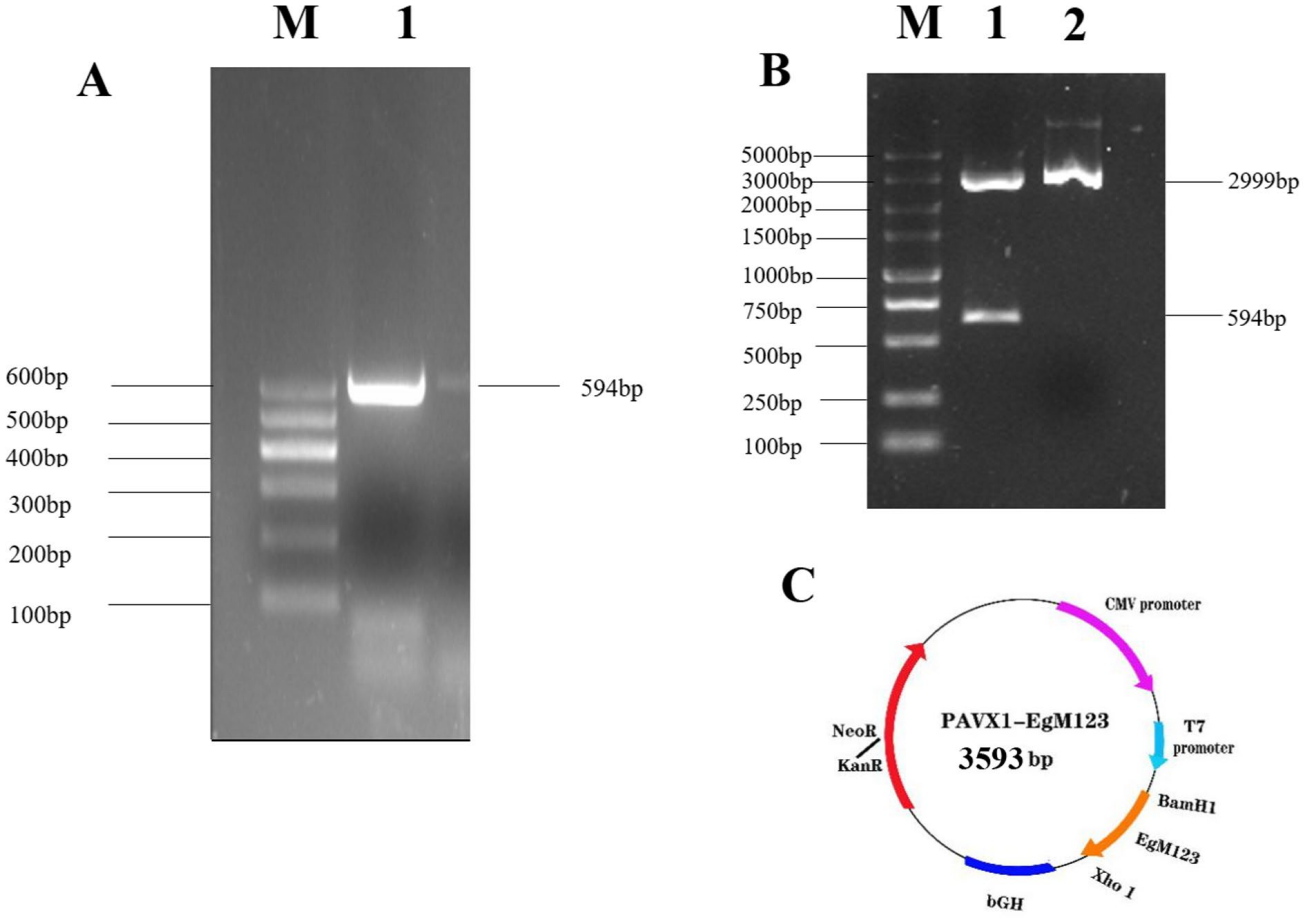
Construction and identification of the recombinant plasmid pVAX1-EgM123. **(A)** The result of PCR amplification of EgM123 gene. Lane M: DNA markers, Lane 1: The PCR amplification of EgM123 gene. **(B)** The result of digestion of the recombinant plasmid pVAX1-EgM123 using the restriction enzymes *BamHI* and *XhoI.* Lane M: DNA markers, Lane 1: The result of digestion of the recombinant plasmid pVAX1-EgM123, Lane 2: The plasmid pVAX1. **(C)** The structure map of the recombinant plasmid pVAX1-EgM123.

The results showed that the open reading frame (ORF) of the EgM123 gene, with a size of 594 bp, was successfully amplified (Figure 1A). The recombinant plasmid pVAX1-EgM123 was digested with *BamHI* and *XhoI*, and subsequent agarose gel electrophoresis confirmed the successful construction of the recombinant plasmid (Figure 1B). The structural map of the recombinant plasmid pVAX1-EgM123 is presented in Figure 1C.

### 3.2 Identifcation of the protein expression of EgM123

**Figure 2:**
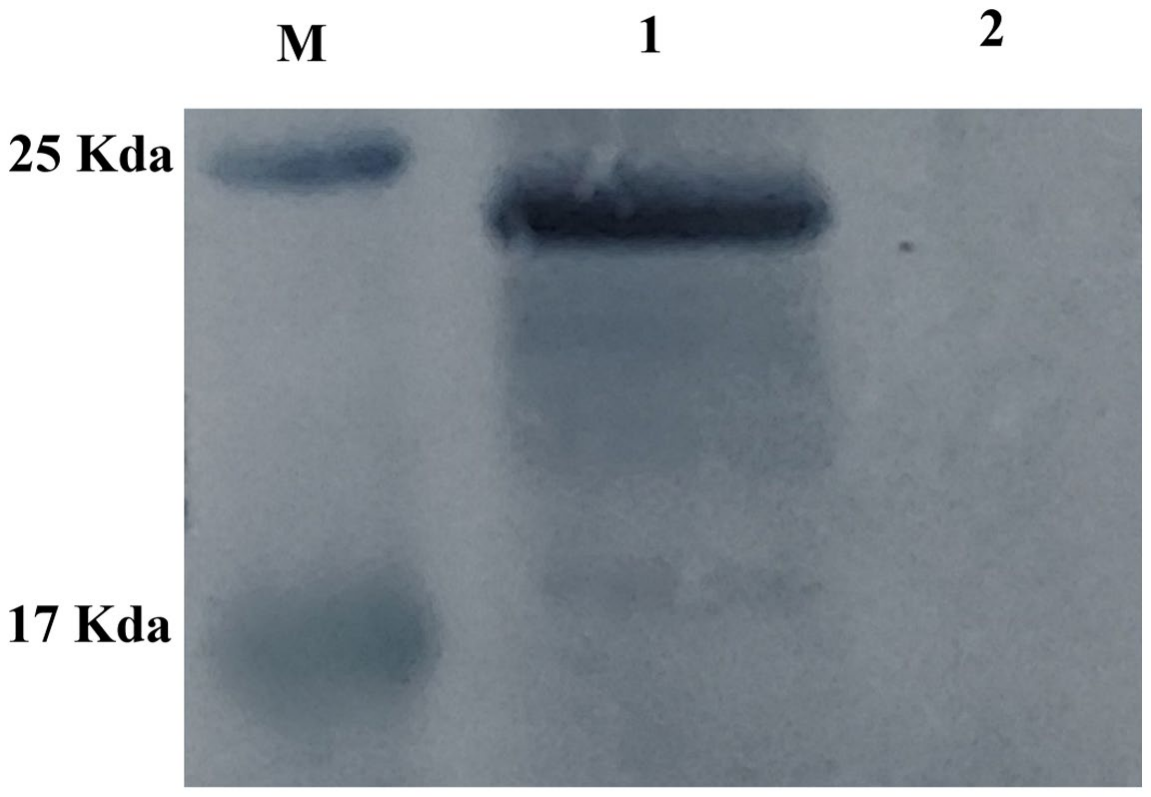
Western blot analysis of the recombinant plasmid pVAX1-EgM123 expression in HEK-293 cells. Lane M: protein markers, Lane 1: The result of the Western blot analysis of the recombinant plasmid pVAX1-EgM123 expression in HEK-293 cells, Lane 2: The result of the Western blot analysis of the plasmid pVAX1 expression in HEK-293 cells.

HEK-293 cells were transfected with either pVAX1-EgM123 or pVAX1 for 48 h, after which the gene expression levels were assessed using Western blotting. The findings revealed the presence of the EgM123 protein (about 22 kDa) in HEK-293 cells transfected with pVAX1-EgM123, while cells transfected with the empty vector did not exhibit any detectable bands (Figure 2). These results suggested that EgM123 proteins was effectively expressed by HEK-293 cells.

### 3.3 Antibody detection

**Figure 3:**
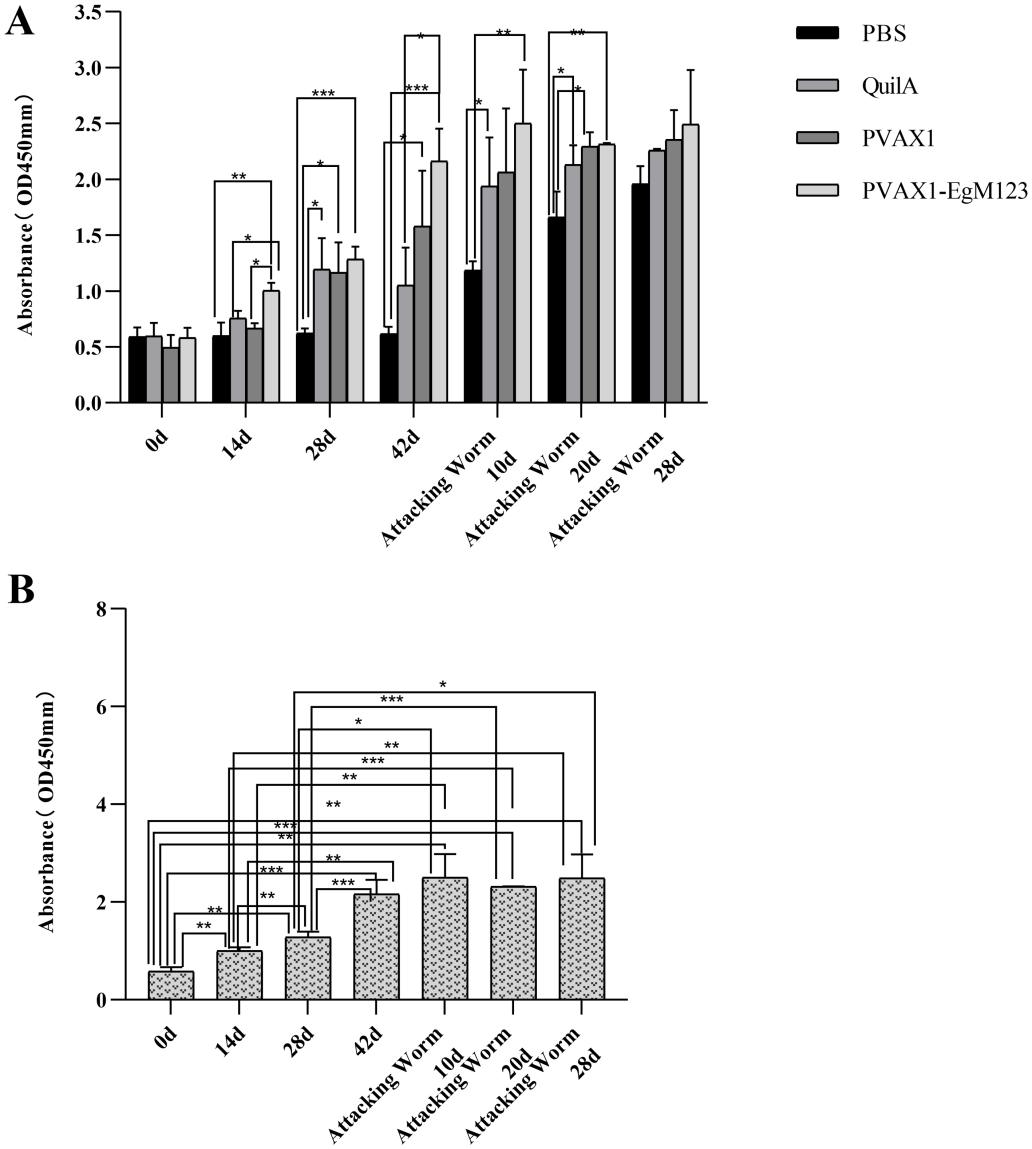
Measurement of specific IgG antibodies in sera of immunized dogs. Sera were collected 1 day prior to each immunization and determined by ELISA. Results are shown as means of OD 490 ± SD and statistical differences are indicated by * compared with PBS, Quil A or pVAX1 (*p < 0.05; **p < 0.01; ***p < 0.001). **(A)** Changes of canine IgG levels in different periods of each group. **(B)** Changes in serum levels of canine IgG at different time points in pVAX1 - EgM123 DNA vaccine group Analysis of variance.

At 14 d, 28 d, 42 d after immunization, 10 d and 20 d after Challenge, the IgG secretion of pVAX1-EgM123 DNA vaccine group was higher than the PBS, QuilA and pVAX1 group. Compared with the PBS group, the IgG in pVAX1-EgM123 DNA vaccine group was significantly increased at 28 d and 42 d (*p* < 0.001) after immunization. At 14 d after immunization, 10 d and 20 d after Challenge, the IgG was also significantly increased (*p* < 0.01). However, there was no significant change at 28 d after Challenge (*p* > 0.05). Compared with the QuilA group, the IgG in pVAX1-EgM123 DNA vaccine group was significantly increased at 14 d, 28 d and 42 d after immunization, 10 d and 20 d after Challenge (*p* < 0.05). There was no significant change at 28 d after challenge (*p* > 0.05). Compared with pVAX1 group, the IgG was significantly increased at 14 d and 42 d after immunization (*p* < 0.05). There were no significant changes at 28 d after immunization, 10 d and 20 d after challenge (*p* > 0.05) (Figure 3A).

The IgG secreted by pVAX1-EgM123 DNA vaccine group at 14 d, 28 d and 42 d after immunization, 10 d, 20 d and 28 d after challenge were compared (Figure 3B). Compared with 0 d, the secretion of the IgG gradually increased at 14 d, 28 d, 42d after immunization, 10 d and 20d after challenge, and reached the highest at 10 d after challenge (*p* < 0.001). Moreover, the IgG increased significantly at 14 d and 28 d after immunization, 10 d and 28 d after challenge (*p* < 0.01). The IgG increased significantly at 28 d after immunization and 20 d after challenge (*p* < 0.001). Compared with 14 d, the IgG was significantly increased at 28 d and 42 d after immunization, 10 d and 28 d after challenge (*p* < 0.01). Compared with 28 d, the IgG was extremely significantly increased at 42 d after immunization and 20 d after challenge (*p* < 0.001). The IgG increased significantly at 20 d and 28 d after challenge (*p* < 0.01). Compared with 42 d, the IgG was significant up-regulated at 0d (*p* < 0.001), 14 d (*p* < 0.01) and 28 d (*p* < 0.001) after immunization. However, there was no significant difference at 10 d, 20 d and 28 d after challenge (*p* > 0.05).

### 3.4 Cytokine measurement

**Figure 4:**
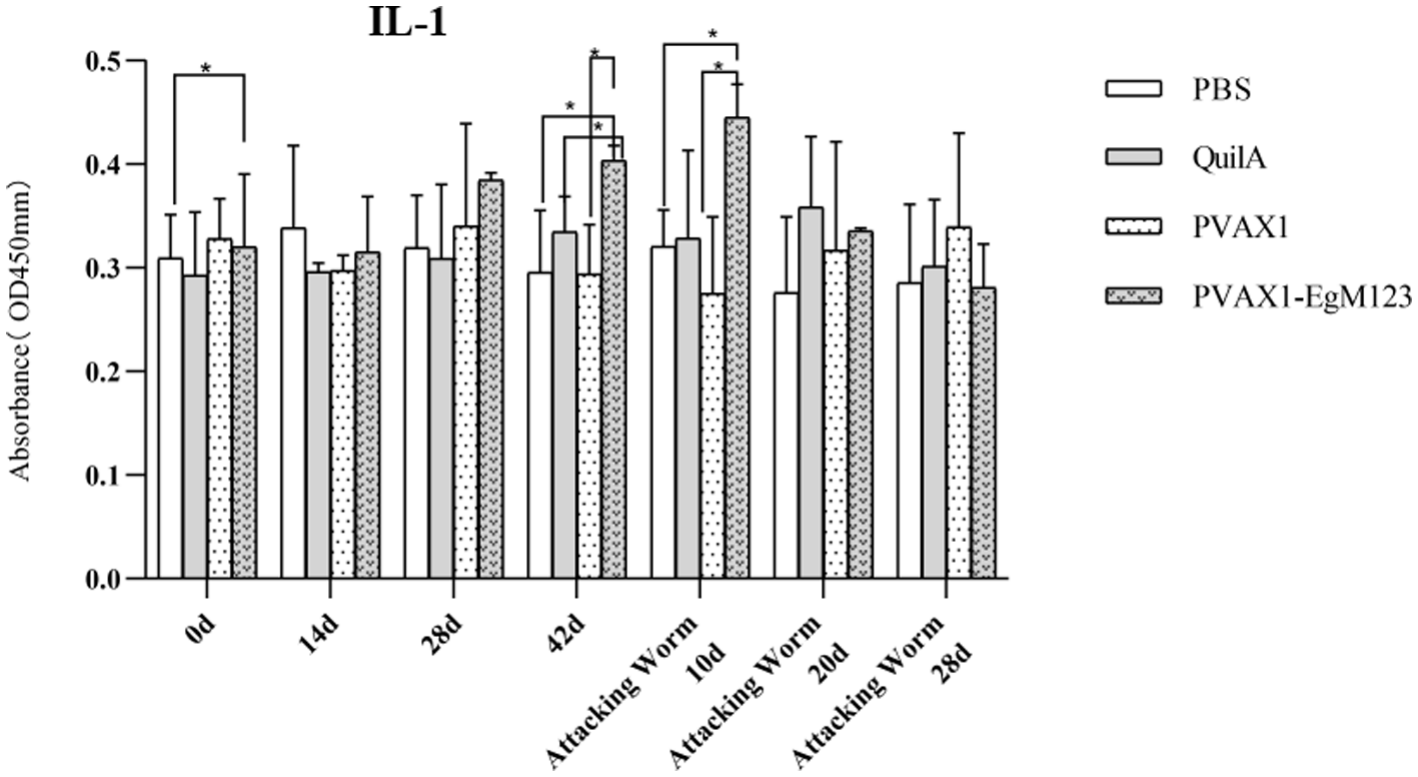
Changes of IL-1 in serum of dogs at different time points. Sera were collected 1 day prior to each immunization and determined by ELISA. Results are shown as means of OD 490 ± SD and statistical differences are indicated by * compared with PBS, Quil A or pVAX1 (*p < 0.05; **p < 0.01; ***p < 0.001).

**Figure 5:**
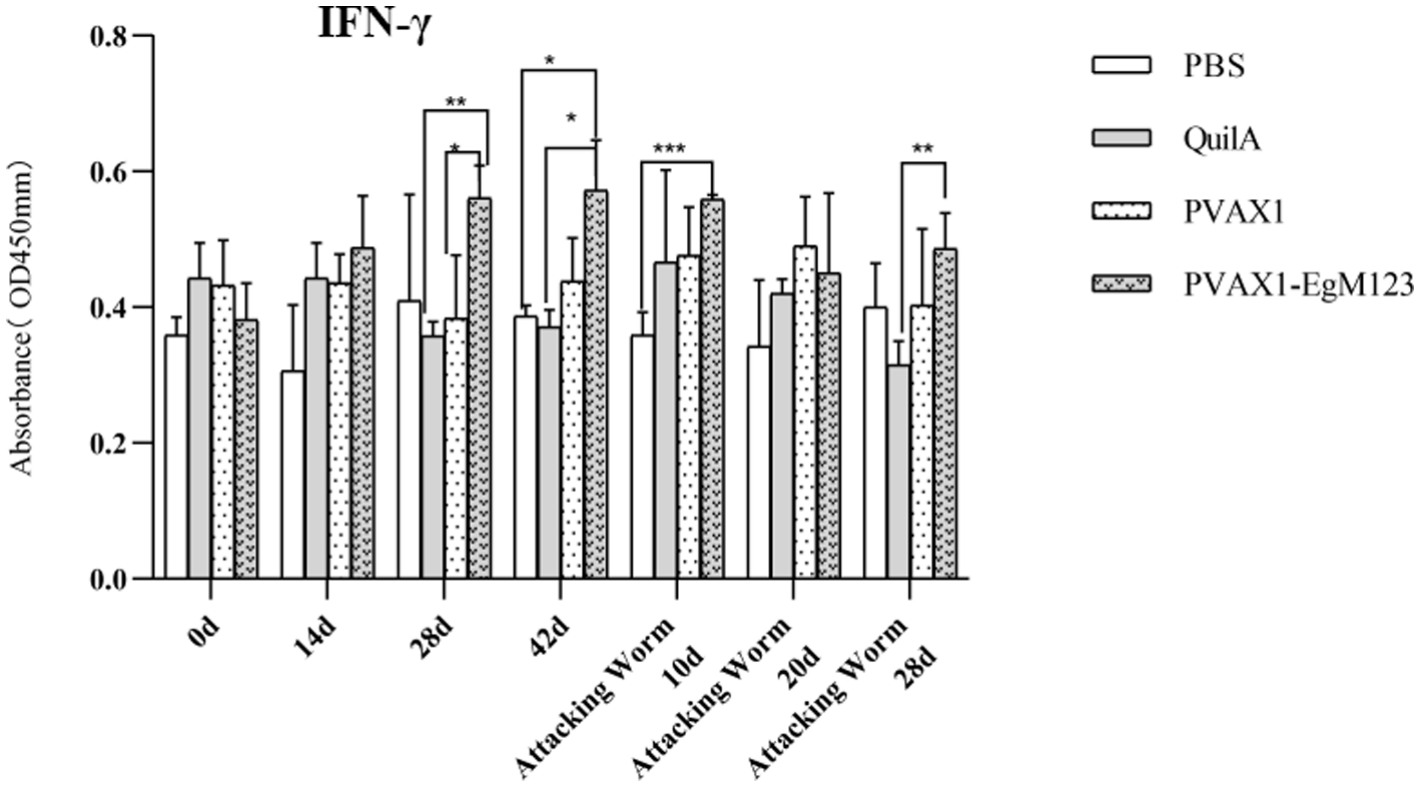
Changes of IFN-*γ* in serum of dogs at different time points. Sera were collected 1 day prior to each immunization and determined by ELISA. Results are shown as means of OD 490 ± SD and statistical differences are indicated by * compared with PBS, Quil A or pVAX1 (*p < 0.05; **p < 0.01; ***p < 0.001).

**Figure 6:**
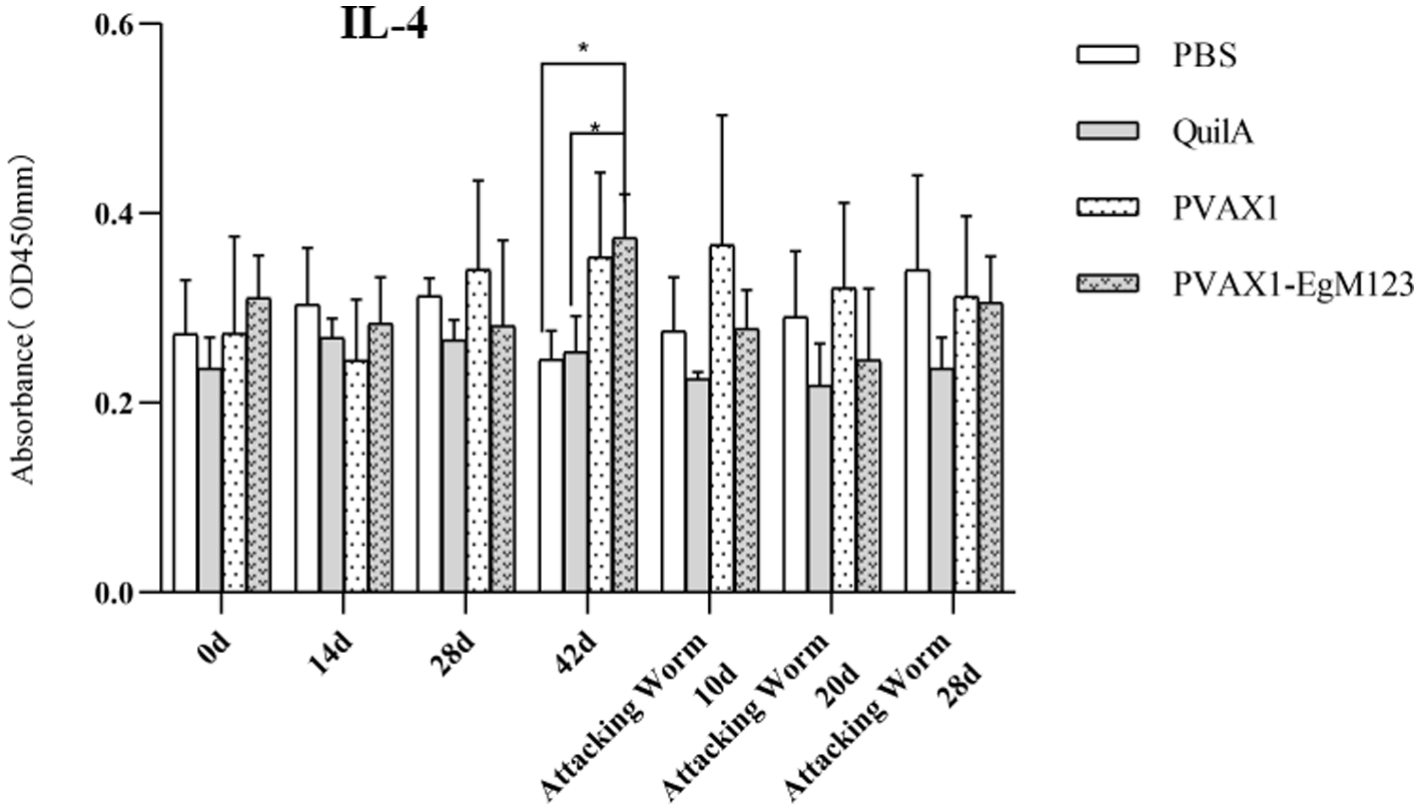
Changes of IL-4 in serum of dogs at different time points. Sera were collected 1 day prior to each immunization and determined by ELISA. Results are shown as means of OD 490 ± SD and statistical differences are indicated by * compared with PBS, Quil A or pVAX1 (*p < 0.05; **p < 0.01; ***p < 0.001).

**Figure 7:**
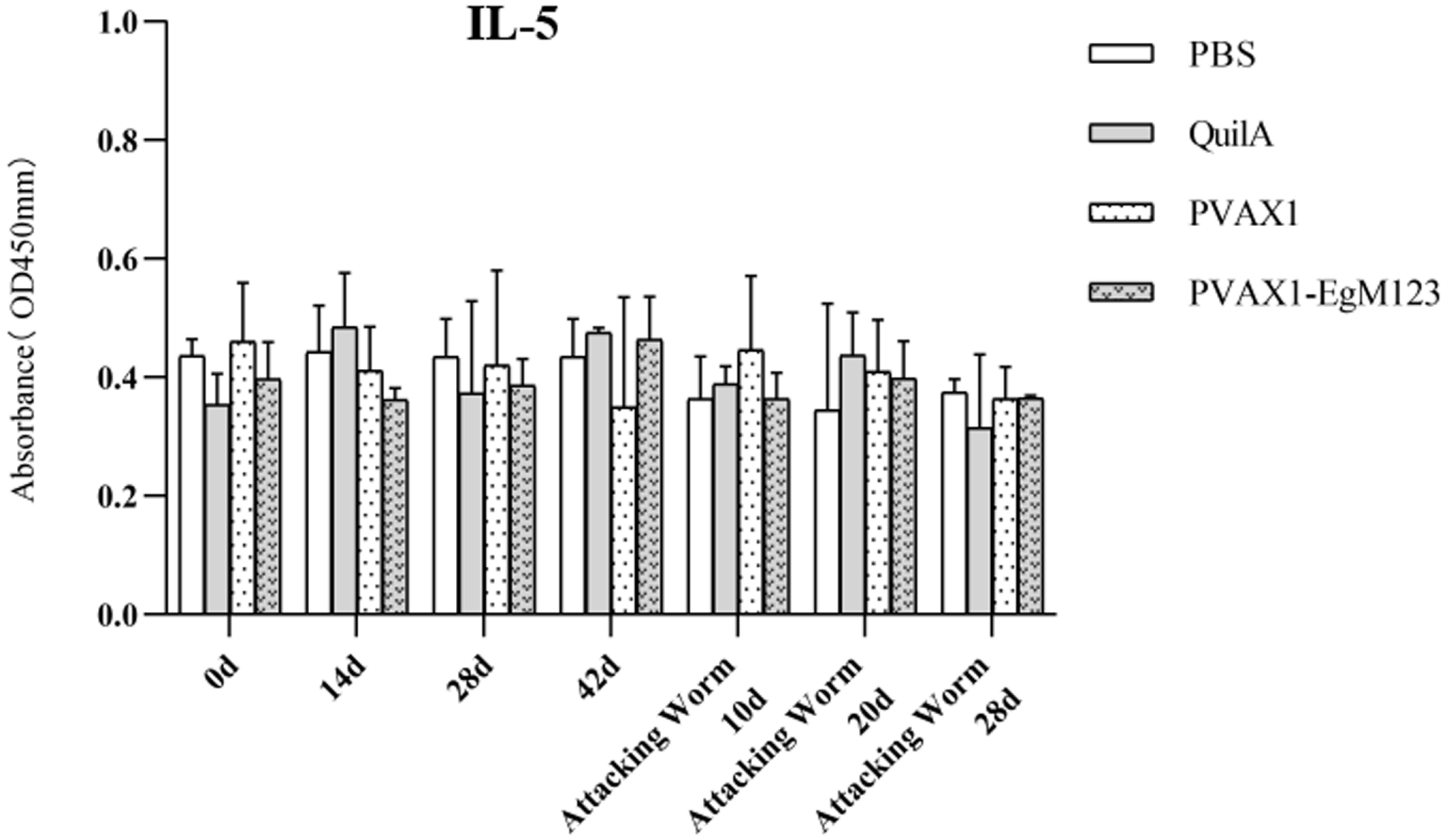
Changes of IL-5 in serum of dogs at different time points. Sera were collected 1 day prior to each immunization and determined by ELISA. Results are shown as means of OD 490 ± SD and statistical differences are indicated by * compared with PBS, Quil A or pVAX1 (* *p* < 0.05, ** *p* < 0.01, *** *p* < 0.001).

**Figure 8:**
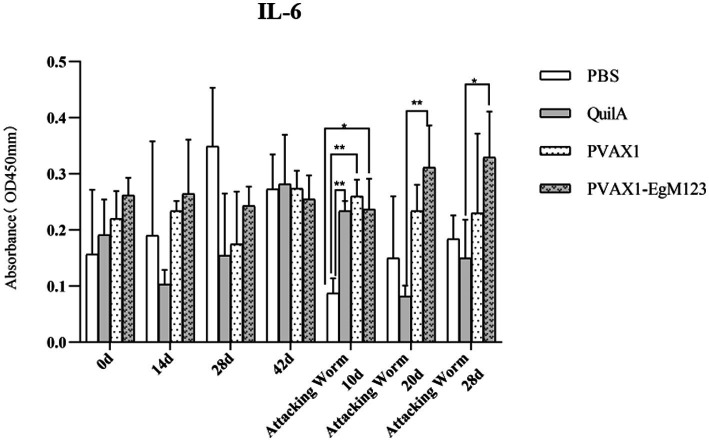
Changes of IL-6 in serum of dogs at different time points. Sera were collected 1 day prior to each immunization and determined by ELISA. Results are shown as means of OD 490 ± SD and statistical differences are indicated by * compared with PBS, Quil A or pVAX1 (*p < 0.05; **p < 0.01; ***p < 0.001).

IL-1, IL-4, IL-5, IL-6 and IFN-*γ* in serum of Beagle dogs at 0d, 14 d, 28 d and 42 d after immunization, 10 d and 20 d after challenge were detected by ELISA. Compared with the PBS group, the pVAX1-EgM123 DNA vaccine group had a significant enhancement effect on IL-1 secretion at 0d and 42d after immunization, 10d after challenge (*p* < 0.05; Figure 4). Moreover, the pVAX1-EgM123 DNA vaccine group significant up-regulated the IFN-*γ* at 42d after immunization (*p* < 0.05) and 10d after challenge (*p* < 0.001; Figure 5). The results also showed that, the IL-4 secretion was significantly enhanced at 42 d after immunization (*p* < 0.05; Figure 6), and the IL-6 secretion was significantly increased at 10 d after challenge (*p* < 0.05). Meanwhile, there was no significant difference in IL-5 secretion (*p* > 0.05; Figure 7). When compared with QuilA group, the IL-1 secretion was significantly increased at 42d after immunization (*p* < 0.05) and 10d after challenge (*p* < 0.05). The IFN-*γ* secretion was significantly increased at 28d and 42d after immunization (*p* < 0.05), and 28d after challenge (*p* < 0.01). The IL-4 secretion was significantly up-regulated at 42d after immunization (*p* < 0.05). The IFN-γ secretion was significantly increased at 28d and 42d after immunization (*p* < 0.05), and 28d after challenge (*p* < 0.01). The IL-6 secretion was significantly up-regulated at 20d (*p* < 0.01) and 28d (*p* < 0.05) after challenge (Figure 8).

### 3.5 Intestinal worm burden and development in dogs

According to statistics, the average worm burden in PBS group was 19,200, in QuilA group was 10,322, in pVAX1 group was 18,222 and in pVAX1-EgM123 DNA vaccine group was 2,333, respectively. Compared the pVAX1-EgM123 DNA vaccine group with the PBS control group, the worm reduction rate of pVAX1-EgM123 DNA vaccine group was 87.85% (*p* > 0.05). Compared the pVAX1-EgM123 DNA vaccine group with the Quil A group, the worm reduction rate of pVAX1-EgM123 DNA vaccine group was 80.26% (p > 0.05). Compared the pVAX1-EgM123 DNA vaccine group with the pVAX1 group, the worm reduction rate of pVAX1-EgM123 DNA vaccine group was 87.20% (*p* < 0.05). Compared the pVAX1 group with the PBS group, the worm reduction rate of pVAX1 group was 5.09% (p > 0.05). Compared the Quil A group with the PBS group, the worm reduction rate of Quil A group was 38.43% (p > 0.05). The results are shown in Table 2.

**Table 2:** Number of Worms in vaccinated dogs after PSC challenge.

Groups	Dogs	Number of worms
PBS	1	36,267
	2	17,333
	3	4,000
Mean ± SD	19,200 ± 9,361	
QuilA	1	26,133
	2	3,467
	3	5,867
Mean ± SD	11,822 ± 7,189	
pVAX1	1	19,200
	2	14,400
	3	21,067
Mean ± SD	18,222 ± 1986	
pVAX1-EgM123	1	1867
	2	2,400
	3	1,066
	4	4,000
Mean ± SD	2,333 ± 619.5	

In order to observe the development of worms, the collected worms of each dog were mixed and poured into a plate, and 30 worms were randomly counted in order in the field of vision. The development of worms was observed, and the number of segments of worms was recorded. According to statistics, the average number of ≤3 segments in PBS group, Quil A group, PXAX1 group and pVAX1-EgM123 DNA vaccine group was 9, 10, 9 and 20, respectively, and ≥ 4 segments were 21, 20, 21 and 10, respectively. Compared the pVAX1-EgM123 DNA vaccine group with the PBS control group, the inhibition rates of PBS group was 30.11% (p<0.05). Compared the pVAX1-EgM123 DNA vaccine group with the QuilA group, the inhibition rates of Quil A group was 30.33% (*p*>0.05). Compared the pVAX1-EgM123 DNA vaccine group with the pXAX1 group, the inhibition rates of pXAX1 group 28.89% (*p*<0.05). The above data indicate that EgM123 DNA vaccine can inhibit the growth and development of *E. granulosus.* The results are shown in Table 3.

**Table 3:** Worm segment development in the experimental groups.

Groups	Dog	The development of worms		
		≤3 segs	≥4 segs	Worm inhibition%
PBS	1	5	25	16.67
	2	13	17	43.33
	3	10	20	30.33
Mean ± SD				30.11 ± 13.33
QuilA	1	3	27	10
	2	9	21	30
	3	18	12	60
Mean ± SD				33.33 ± 25.17
pVAX1	1	7	23	23.33
	2	11	19	36.67
	3	8	22	26.67
Mean ± SD				28.89 ± 6.94
pVAX1-EgM123	1	23	7	76.67
	2	16	14	53.33
	3	15	15	50.00
	4	24	6	80.00
Mean ± SD				65.00 ± 15.52

## 4 Discussion

Current studies on intermediate host vaccines against *E. granulosus* include natural worm crude antigens and recombinant protein antigens. The natural worm crude antigens include the oncosphere, germinal layer, and protoscoleces antigens (20–23). The recombinant protein antigens includes AgB (24, 25), EpC1 (26) and so on. Up to now, the most successful intermediate host vaccine is the Eg95 recombinant protein with a protective efficiency of up to 98% (27). However, recombinant Eg95 protein has no protective effect on hosts who have been infected with *E. granulosus* and formed cysts. Conversely, it is worth noting that the population of definitive hosts, primarily dogs, is significantly smaller compared to that of intermediate hosts, such as cattle and sheep. Consequently, vaccinating the definitive hosts would result in a substantial reduction in the overall cost of immunization. Unfortunately, there is no commercial definitive-host vaccine yet. Therefore, the development of a dog vaccine against *E. granulosus* is urgently needed. Of course, there are many studies related to vaccine candidate antigens for the definitive hosts against *E. granulosus* infection. Notably, antigens such as EgA31 (19, 28, 29), P-29 (30), EgG1Y162 (29, 31), Myophilin (32), Tropomyosin (33), EgM family (10, 11), EgHCDH (16), TSP11 (17) and ANX (34) have demonstrated partial immunity and protective effects for definitive hosts. All these data provide theoretical basis for the successful development of vaccines against *E. granulosus* in the future.

IgG, comprising approximately 80% of the total serum immunoglobulin, is the predominant antibody component found in both serum and body fluid. This immunoglobulin is extensively distributed throughout the body and plays a crucial role in immune responses. In this study, the researchers observed the alteration in the overall IgG levels present in the serum of dogs. The findings indicated that the administration of the pVAX1-EgM123 DNA vaccine prompted the production of targeted antibodies in the dogs, resulting in an increase in IgG concentration within their serum. This increase was observed to be directly proportional to the number of immunizations received, reaching its peak at 10 days post-challenge. Our study revealed that the PVAX-EgM123 DNA vaccine elicited a similar change in canine antibody levels as the recombinant protein vaccine of the EgM family (10, 11). Additionally, we observed a partial similarity between the effects of the pVAX1-EgM123 DNA vaccine on canine antibody levels and the TgGRA16 DNA vaccine of Toxoplasma on its host antibody levels (35). The secretion of total IgG antibodies in the pVAX1-EgM123 DNA vaccine group was found to be significantly increased after the second and third immunization compared to the PBS group (*p* < 0.001). Similarly, when compared to the QuilA group, the secretion of total IgG antibodies was significantly increased after the first, second, third, 10 days, and 20 days after infection (*p* < 0.05). Furthermore, compared to the pVAX1 group, the secretion of total IgG antibodies was significantly increased after the primary and third immunization (*p* < 0.05). The findings of this study demonstrated that the pVAX1-EgM123 DNA vaccine elicited the production of targeted antibodies in canines.

Research has indicated that following infection with *E. granulosus*, the host exhibits two primary immune responses: Th1-type and Th2-type. Th1 immune responses have been found to impede the parasite’s development, growth, and pathogenicity within the host. Conversely, the Th2 immune response is capable of facilitating humoral response and exerting an immunosuppressive effect, thereby hindering the timely eradication of pathogens and allowing parasitic pathogens to persist within the host organism for extended durations (36). In most cases, the Th1 and Th2 immune responses coexist and regulate each other (37). In the majority of cases, the coexistence and reciprocal regulation of Th1 and Th2 immune responses have been observed (37). In this particular study, the levels of Th1 and Th2 related cytokines were measured separately. The findings revealed that, compared to the control group receiving PBS, the group vaccinated with pVAX1-EgM123 DNA exhibited a significant increase in IL-1 secretion at 0 days and 42 days post-immunization, as well as 10 days after challenge (*p* < 0.05). Furthermore, the pVAX1-EgM123 DNA vaccine group demonstrated a significant up-regulation of IFN-*γ* at 42 days post-immunization (*p* < 0.05) and 10 days after challenge (*p* < 0.001). The findings align with previous research on the efficacy of ROP5 and ROP18 cocktail DNA vaccines in conferring protective immunity against *Toxoplasma gondii* infection in mice (38). These results indicate that the pVAX1 - EgM123 DNA vaccine effectively stimulates Th1 immune responses in canines, potentially serving as a crucial factor in impeding worm development and facilitating worm elimination by the host. Furthermore, in comparison to the control group, the pVAX1-EgM123 DNA vaccine group exhibited a noteworthy augmentation in the secretion of IL-4 42 days post-immunization (*p* < 0.05), as well as a significant elevation in IL-6 secretion 10 days after challenge (*p* < 0.05). These findings strongly suggest that the pVAX1-EgM123 DNA vaccine elicits Th2 immunity in canines. Consequently, these results provide evidence that the pVAX1-EgM123 DNA vaccine is capable of inducing both Th1 and Th2 immune responses in dogs. The results were consistent with Rashid’s study, demonstrating that the ROP18 DNA plasmid possesses the capacity to elicit both Th1 and Th2 immune responses in the host in response to chronic toxoplasmosis (39).

Skeletal muscle represents the optimal cellular substrate for gene immunotherapy due to its ease of administration via injection. Additionally, the abundant presence of blood vessels within skeletal muscle facilitates efficient systemic dissemination of therapeutic targets into the circulatory system (40). The introduction of DNA vaccines into skeletal muscle cells has been shown to augment both humoral and cellular immune responses (41). The semitendinosus muscle in canines is situated posteriorly to the gluteus biceps, originating from the sciatic tubercle and terminating at the tibial ridge, calf fascia, and calcaneus tubercle. Its primary functions include extension of the hip joint, as well as flexion of the knee and tarsal joints. Additionally, the semimembranosus muscle is located in the posterior medial region of the semitendinosus, originating from the sciatic tubercle and concluding at the medial aspect of the distal femur. Its main actions involve hip joint extension and hind limb retraction. The semitendinosus and semimembranous muscles, which are part of the posterior femoral muscle group, exhibit significant development. In their study, Arce-Fonseca et al. investigated the immune response elicited by the pBCSSP4 DNA vaccine against *Trypanosoma graminis*. Specifically, they administered 500 μg of the pBCSSP4-DNA vaccine plasmid to Beagle dogs via the semitendinosus and semimembranous muscles. The findings of the study demonstrated that pBCSSP4 elicited both Th1 and Th2 immune responses, resulting in a reduction in heart damage (42). Moreover, the study also proposed the effectiveness of administering the DNA vaccine via the semitendinosus and semimembranosus muscles. Building upon these premises, the current investigation employed an identical immune dosage and pathway.

The EgM family has been initially recognized as a promising antigen for the development of a vaccine against *E. granulosus* infection in canines. In a study conducted by Zhang et al. in 2006, dogs were immunized with recombinant EgM4, EgM9, and EgM123, resulting in notable protective effects. These three proteins exhibited significant efficacy in reducing worm burden and impeding worm development. Particularly, EgM123 demonstrated an impressive reduction rate of 92%, with only 3.5% of the worms progressing to the adult stage, and no ovulation worms were observed 45 days post-infection (10). Subsequently, the research team administered a combination of EgM9 and EgM123 recombinant proteins, along with QuilA, to immunize dogs. The findings revealed a notable reduction rate of 89.2% in worm burden after a 45-day challenge period, with no observation of mature or ovulated worms. These results indicate that the amalgamation of EgM123 recombinant protein and QuilA exerts a substantial inhibitory impact on worm growth and fecundity (11). In this study, the pVAX1-EgM123 DNA vaccine was combined with QuilA, and the findings indicated that the pVAX1-EgM123 DNA vaccine group exhibited a worm reduction rate of 87.85% at 28 days post-challenge, along with a segments inhibition rate of 65.00%. These results suggest that the pVAX1-EgM123 DNA vaccine demonstrates a comparable immune response to the recombinant EgM123 protein in terms of worm reduction rate. However, it exhibits a lower inhibitory effect on the growth and development of worms compared to the recombinant EgM123 protein. We speculate that the observed outcome May be attributed to the exclusive composition of plasmids in DNA vaccines, in contrast to recombinant protein vaccines. These plasmids elicit an immune response by expressing the antigen genes they bear upon transfection into cells (43). In recent years, a substantial body of research has consistently revealed that multiple factors exert an influence on the immune response evoked by DNA vaccines. The selection of the candidate antigen and the eukaryotic expression vector hold particular importance in the advancement of DNA vaccines. It is crucial for vaccine candidates to effectively elicit both protective cellular Th1 and humoral Th2 responses, both at the intestinal mucosa and throughout the entire organism (44). In this study, the selection of EgM123 was based on its immunodominance. Moreover, the pVAX1 co-expression vector was chosen for the construction of the DNA vaccine due to its possession of multiple restriction sites, allowing for the simultaneous cloning of extensive segments of the target gene. Additionally, the presence of the robust PCMV promoter and BGH poly a signal within the vector facilitates the expression of recombinant proteins at high levels in mammalian cells, rendering it a suitable option for the construction of a DNA vaccine for mammals species.

In summary, this research presents preliminary results regarding the application of a DNA vaccine directed against *Echinococcus granulosus* in dogs. The pVAX1-EgM123 DNA vaccine plasmid was successfully constructed and delivered to a cohort of Beagle dogs. The immune response to the pVAX1-EgM123 DNA vaccine was assessed by measuring changes in antibody concentrations in canine serum, cytokine production, parasite load, and parasite development.The findings of this study suggest that the pVAX1-EgM123 DNA vaccine induces an immunogenic response in dogs, marked by the generation of specific antibodies and a Th1/Th2 immune reaction. Additionally, the vaccine exhibits effectiveness in diminishing worm load and impeding parasite development in experimental canines. These results propose that the pVAX1-EgM123 DNA vaccine shows potential as a prospective remedy for managing *E. granulosus* infection in dogs.

## Data Availability

The original contributions presented in the study are included in the article/supplementary material, further inquiries can be directed to the corresponding author/s.
